# Supplementary figures and images for: Non-destructive prediction of anthocyanin concentration in whole eggplant peel using hyperspectral imaging
Source: PeerJ. 2024 May 14;12:e17379. doi: 10.7717/peerj.17379 (PMC11636719; doi:10.7717/peerj.17379)

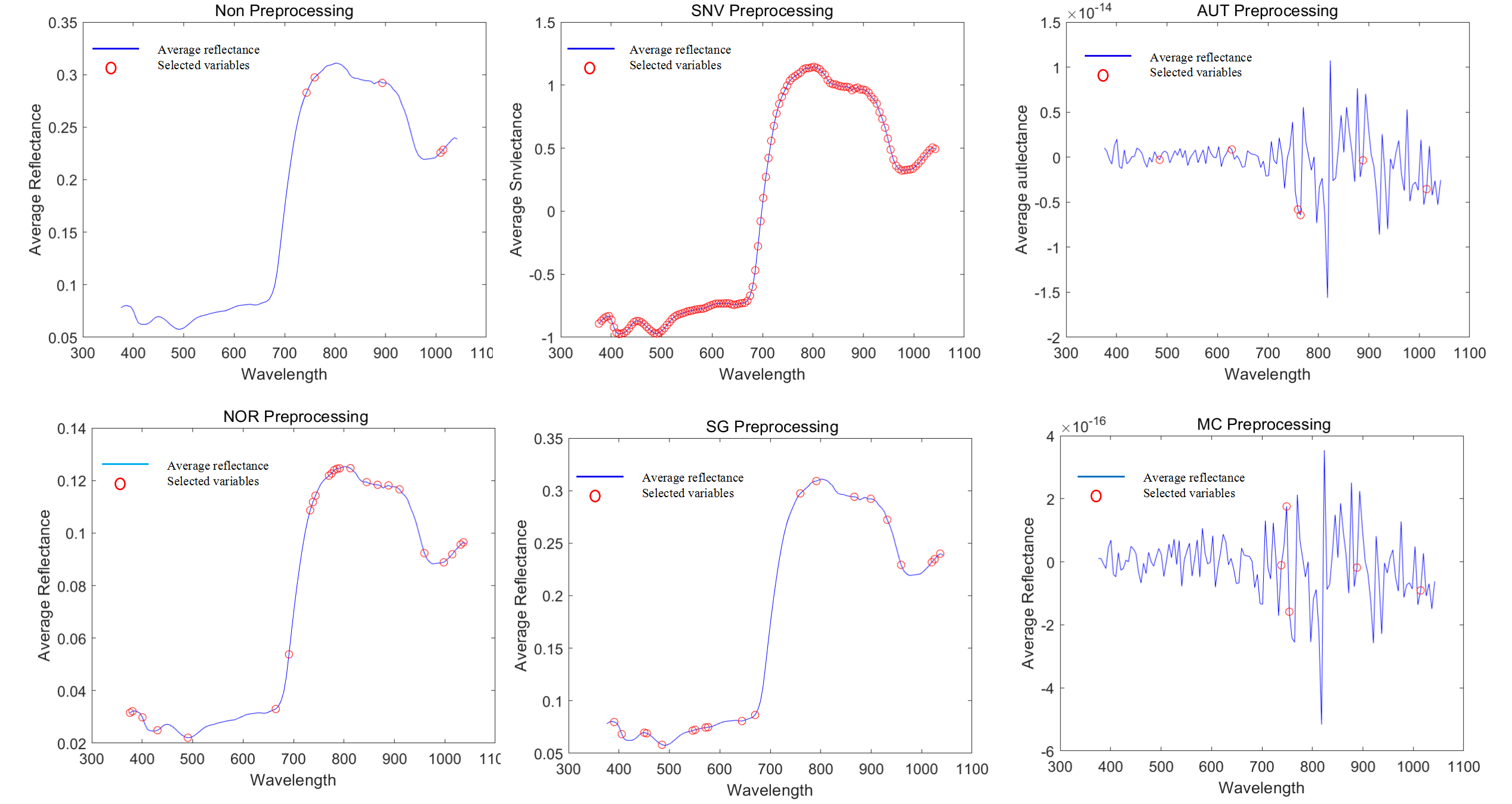

Supplement: Supplemental Information 3 [file peerj-12-17379-s003.png]

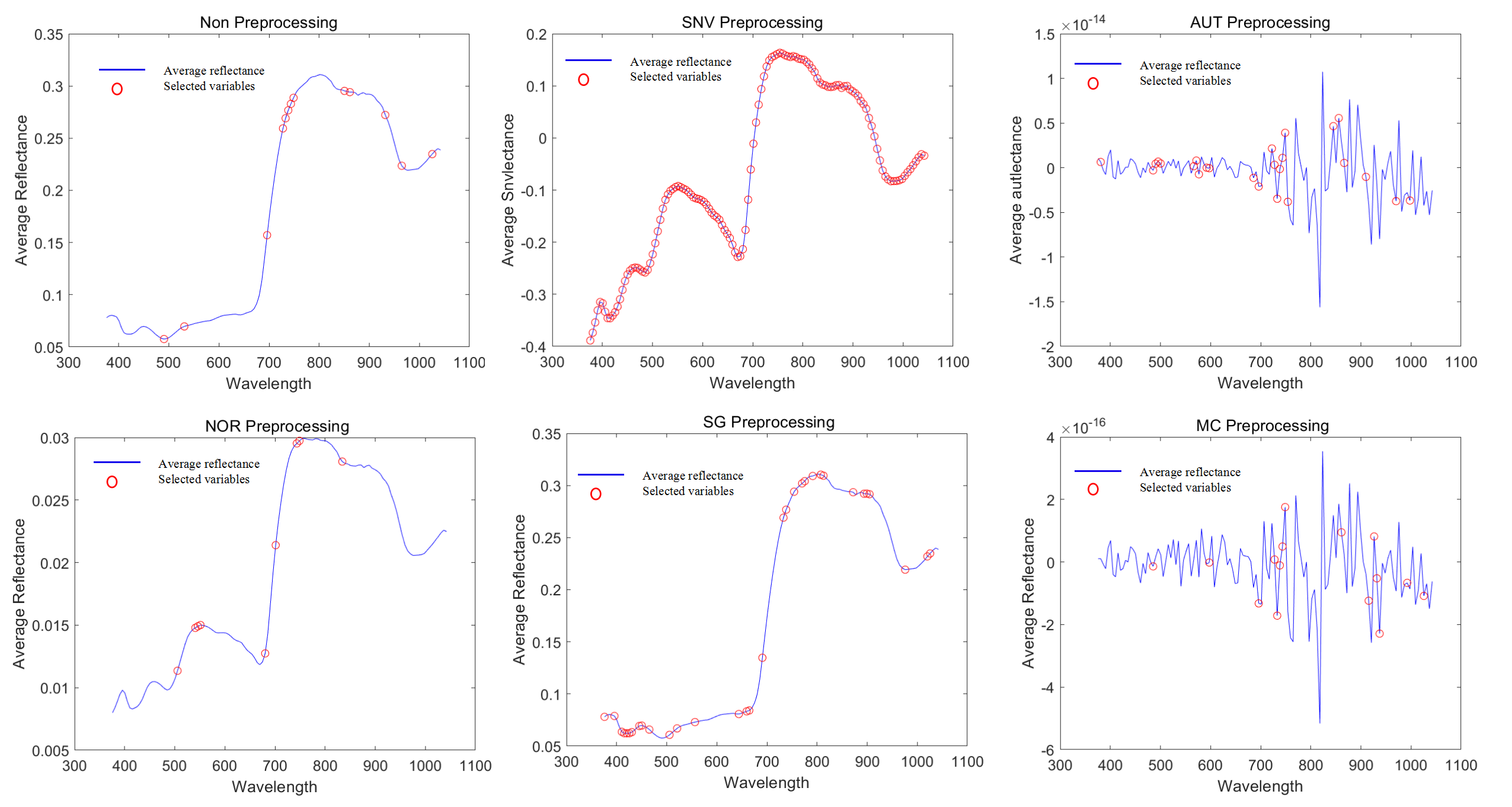

Supplement: Supplemental Information 4 [file peerj-12-17379-s004.png]

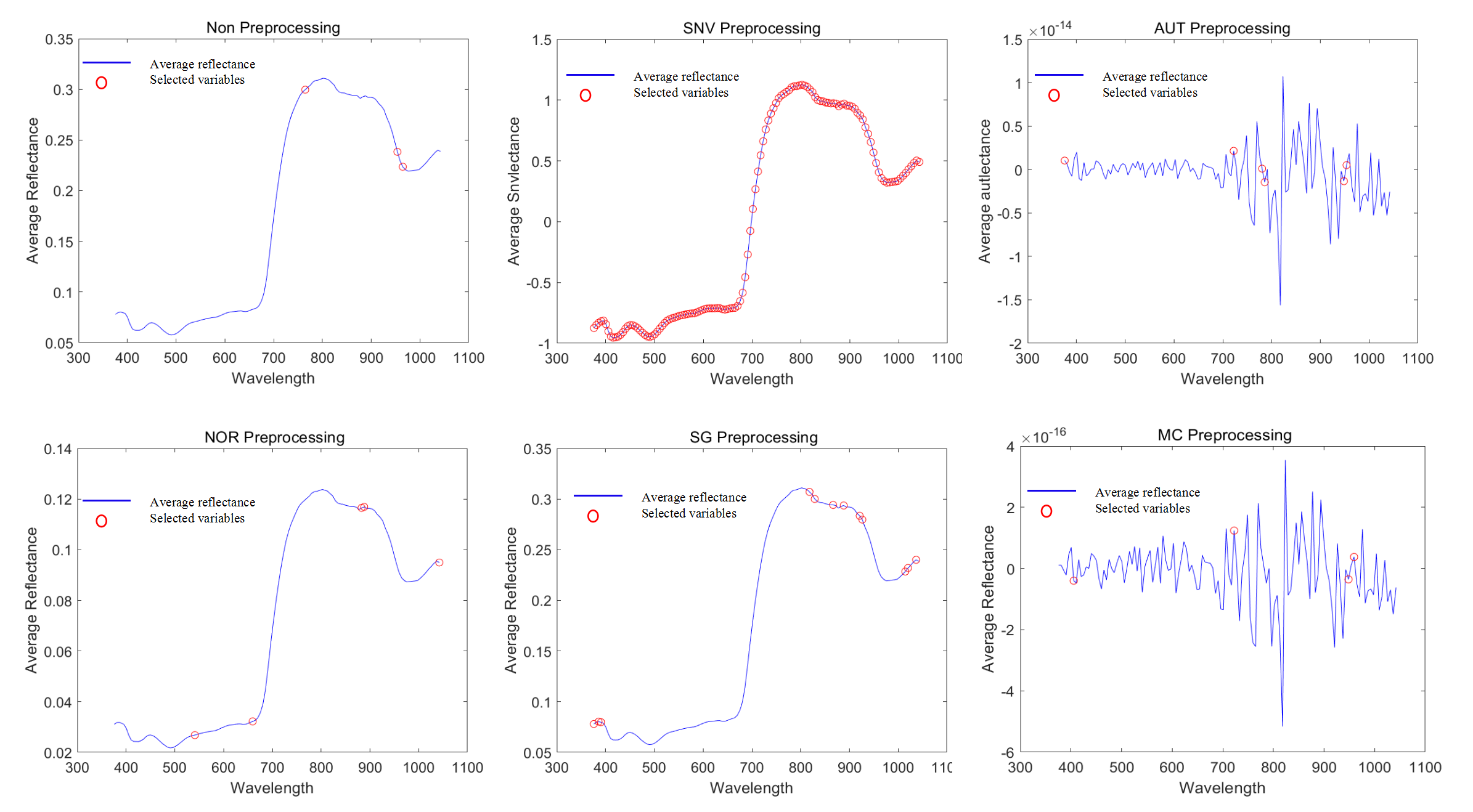

Supplement: Supplemental Information 5 [file peerj-12-17379-s005.png]

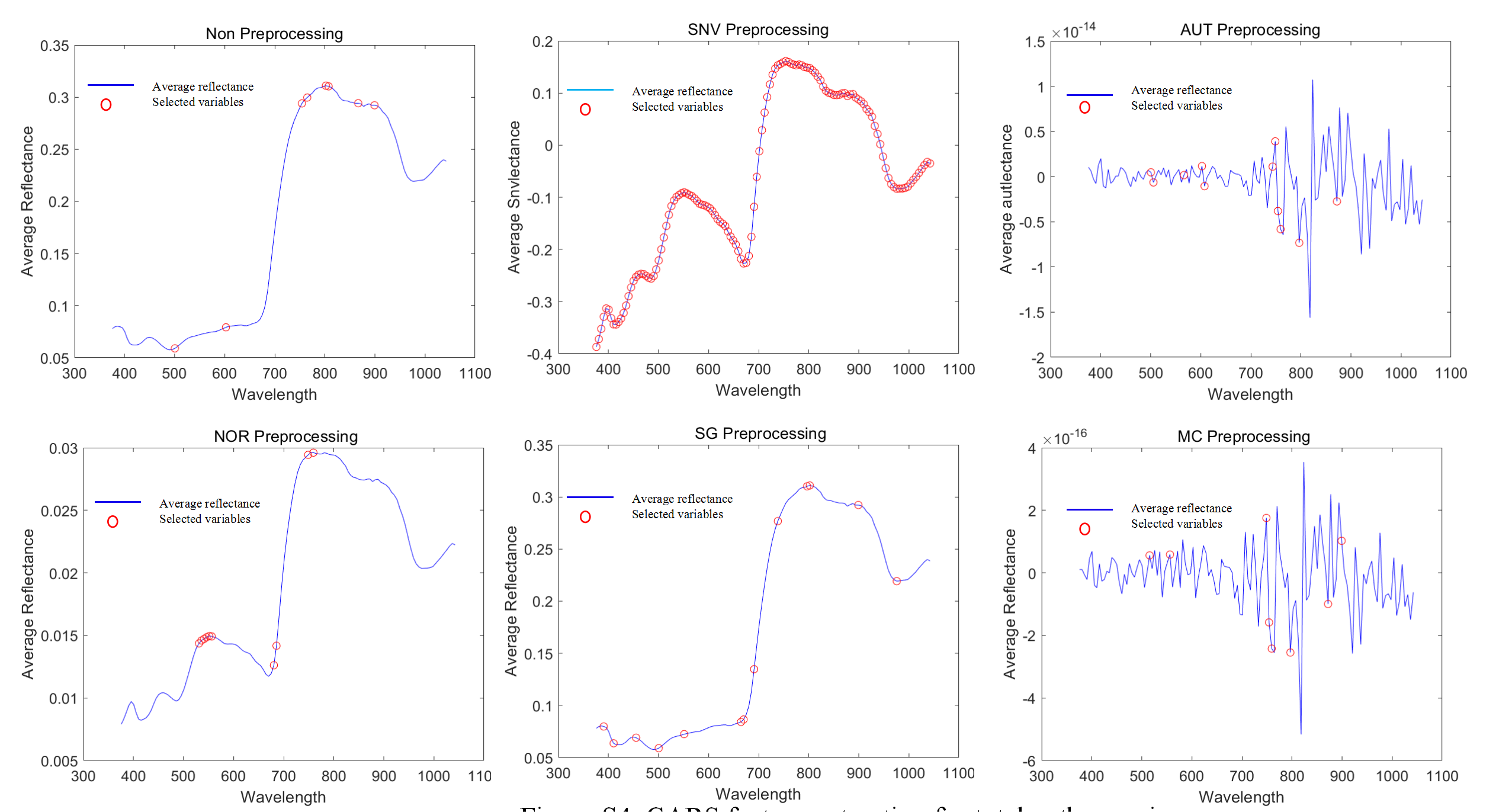

Supplement: Supplemental Information 6 [file peerj-12-17379-s006.png]
